# Allopregnanolone pleiotropic action in neurons and astrocytes: calcium signaling as a unifying mechanism

**DOI:** 10.3389/fendo.2023.1286931

**Published:** 2023-12-22

**Authors:** Tian Wang, Shuhua Chen, Zisu Mao, Yuan Shang, Roberta Diaz Brinton

**Affiliations:** ^1^ Center for Innovation in Brain Science, University of Arizona, Tucson, AZ, United States; ^2^ Department of Neurology, College of Medicine Tucson, University of Arizona, Tucson, AZ, United States; ^3^ Department of Pharmacology, College of Medicine Tucson, University of Arizona, Tucson, AZ, United States

**Keywords:** allopregnanolone, calcium signaling, mitochondria, neuroplasticity, astrocytic function

## Abstract

**Objective:**

Allopregnanolone (Allo) is a neurosteroid with pleiotropic action in the brain that includes neurogenesis, oligogenesis, human and rodent neural stem cell regeneration, increased glucose metabolism, mitochondrial respiration and biogenesis, improved cognitive function, and reduction of both inflammation and Alzheimer’s disease (AD) pathology. Because the breadth of Allo-induced responses requires activation of multiple systems of biology in the absence of an Allo-specific nuclear receptor, analyses were conducted in both neurons and astrocytes to identify unifying systems and signaling pathways.

**Methods:**

Mechanisms of Allo action were investigated in embryonic hippocampal neurons and astrocytes cultured in an Aging Model (AM) media. Cellular morphology, mitochondrial function, and transcriptomics were investigated followed by mechanistic pathway analyses.

**Results:**

In hippocampal neurons, Allo significantly increased neurite outgrowth and synaptic protein expression, which were paralleled by upregulated synaptogenesis and long-term potentiation gene expression profiles. Mechanistically, Allo induced Ca^2+^/CREB signaling cascades. In parallel, Allo significantly increased maximal mitochondrial respiration, mitochondrial membrane potential, and Complex IV activity while reducing oxidative stress, which required both the GABA_A_ and L-type Ca^2+^ channels. In astrocytes, Allo increased ATP generation, mitochondrial function and dynamics while reducing oxidative stress, inflammasome indicators, and apoptotic signaling. Mechanistically, Allo regulation of astrocytic mitochondrial function required both the GABA_A_ and L-type Ca^2+^ channels. Furthermore, Allo activated NRF1-TFAM signaling and increased the DRP1/OPA1 protein ratio, which led to increased mitochondrial biogenesis and dynamics.

**Conclusion:**

Collectively, the cellular, mitochondrial, transcriptional, and pharmacological profiles provide evidence in support of calcium signaling as a unifying mechanism for Allo pleiotropic actions in the brain.

## 1 Introduction

Allopregnanolone (Allo, 3α-hydroxy-5α-pregnan-20-one) is an endogenous neurosteroid derived from progesterone (1–6). Despite the lack of a specific nuclear transcriptional receptor, Allo functions as a pleiotropic systems biology regulator (1–3, 7–12). The systems of biology activated by Allo in the brain include neural stem cell regeneration, neurogenesis, oligogenesis and increased white matter (3, 7, 13–19), mitochondrial respiration and bioenergetics (10, 20), cholesterol homeostasis (21, 22), immune regulation (12, 21, 23–25), reduced beta amyloid and pTau (21), restoration of synaptic transmission, and cognition (9, 26, 27).

The mechanisms of action of Allo have been extensively studied in mature neurons and neural stem cells (3, 13, 28–30). In both cell types, Allo prolongs the opening of the GABA_A_ receptor complex (30). However, modulation of the GABA_A_ receptor complex by Allo results in hyperpolarization in mature neurons and the opposite, depolarization, in neural stem cells (3, 13). Mature neurons, although expressing Na-K-Cl cotransporter (NKCC1), maintain a low intracellular Cl^−^ level due to the upregulation of the neuron-specific K-Cl cotransporter 2 (KCC2) during their maturation (31). Allo prolongation of the GABA_A_ receptor complex open time increases chloride influx, hyperpolarization and potentiation of the inhibitory post-synaptic potential (30). In contrast, neural stem cells exhibit a high-intracellular Cl^−^ level attributed to NKCC1 expression (3). Allo prolongs the GABA_A_ receptor complex open time, which results in depolarization of the plasma membrane due to efflux of intracellular chloride (3, 32). This Allo-induced membrane depolarization leads to activation of voltage-dependent L-type calcium (Ca^2+^) channels (28). The subsequent rise in intracellular Ca^2+^ activates a Ca^2+^-dependent kinase, CaMK IV, which then phosphorylates and activates the transcription factor cyclic AMP-responsive element-binding protein 1 (CREB1) (3). Through CREB1 activation, Allo upregulates the expression of cell cycle genes that promote mitosis, leading to increased neural stem cell regeneration (3, 13, 28).

The breadth of Allo regulation of systems of biology requires multiple cell types. Although the effects of Allo have been reported in multiple neural cell types (33), a unifying mechanism of action across multiple cell types remains unresolved. Herein, we investigated the impact of Allo on a common set of responses in embryonic hippocampal neurons and astrocytes, both of which express GABA_A_ receptors and have a high-intracellular chloride concentration (31, 34, 35). Transcriptional analyses were conducted to identify both unique and common pathways relevant to Allo-induced outcomes.

Results reported herein indicate that, in embryonic neurons, Allo activates the Ca^2+^/CREB signaling pathway that promotes neural plasticity and mitochondrial function. In parallel, Allo activated the Ca^2+^ signaling pathway in astrocytes, which was associated with increased mitochondrial biogenesis and dynamics through an increase in the NRF1-TFAM signaling cascade and DRP1/OPA1 protein ratio. These mitochondrial outcomes were associated with reduced inflammasome activation and oxidative stress. Notably, Allo’s systems of biology actions rely on a high-intracellular Cl^−^ concentration, mediated by expression of the NKCC1, that enables efflux of intracellular Cl^−^ through the GABA_A_ receptor and thus membrane depolarization that activates the L-type Ca^2+^ channels enabling an influx of Ca^2+^ and activation of the Ca^2+^ signaling pathway (3). Collectively, findings reported herein provide insights into common Ca^2+^-mediated and mitochondria-related mechanisms that underlie the pleiotropic action of Allo to activate systems of biology enabling integration of signaling and function in neurons and astrocytes.

## 2 Materials and methods

### 2.1 Isolation of primary hippocampal neurons and astrocytes

Primary embryonic hippocampal neurons and astrocytes were isolated from day 18 (E18) fetuses of Sprague Dawley rats (Envigo) as previously described (36, 37). Briefly, hippocampi were dissected from the brains of E18 fetuses and digested by incubation with 0.02% trypsin in HBSS (Invitrogen,14170-112) for 3 min at 37°C. The digested tissue was dissociated by repeated passing through a series of fire-polished constricted Pasteur pipettes, then filtered through a 40-µm Nylon cell strainer into 50-ml conical centrifuge tubes and centrifuged at 1,000 rpm for 3 min.

Neurons were cultured in Neurobasal Medium (Gibco, 12348017) supplemented with 2%B-27 (Gibco, 17504044), 25 μM glutamate (MP Biomedicals, 02101800), 0.5 mM L-glutamine (Gibco, 25030081) and 10U/mL penicillin/streptomycin (Gibco, 15140122). Mixed glia was cultured in Dulbecco’s Modified Eagle Medium (DMEM)/F-12 (Gibco, 11039021) supplemented with 10% FBS (ATCC, 30-2020) and 20 U/mL penicillin/streptomycin (Gibco, 15140122) in T75 flasks. Upon 80% confluency, cultures were shaken on an orbital shaker overnight and then washed with cold Phosphate buffer saline (PBS). The supernatants containing microglia and oligodendrocytes were discarded, and the remaining enriched astrocytes were then trypsinized and re-plated onto poly-D-lysine coated wells or coverslips.

For imaging or immunocytochemistry studies, 20,000 cells were plated onto each poly-D-lysine (50 μg/mL) coated 22-mm coverslip. For Western blotting, 10^6^ cells were seeded on poly-D-lysine coated six well plates. Cells were incubated at 37°C in a humidified 5% CO_2_ incubator.

### 2.2 In-vitro treatment

#### 2.2.1 Allo treatment

**Figure 1:**
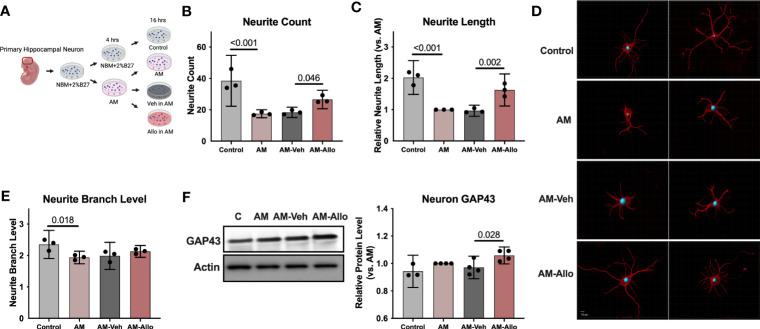
Allo treatment significantly reversed AM-induced deficits in neurite outgrowth. **(A)** The schematic diagram of Allo treatment (Created with BioRender.com). Allo treatment (100 nM) reversed AM-induced deficits in neurite count **(B)** (*n* = 3, one-way analysis of variance (ANOVA), F (3, 8) = 22.28, *p* < 0.001, η^2 ^ = 0.893) and neurite length **(C)** (*n* = 3, one-way ANOVA, F (3, 8) = 33.38, *p* < 0.001, η^2^ = 0.926). **(D)** Representative neuron images labeled with anti–β-tubulin antibody and processed with Imaris. **(E)** The significant decrease in neurite branch levels induced by AM (*n* = 3, one-way ANOVA, F (3, 8) = 5.491, *p* = 0.024, η^2^ = 0.673) was not affected by Allo treatment. **(F)** Relative to AM-Veh group, AM-Allo treatment significantly increased GAP43 protein levels (*n* = 3–4, one-way ANOVA, F (3, 11) = 5.763, *p* = 0.013, η^2^ = 0.611). All bar graphs are presented as mean ± 95% CI with individual data points. Statistical significance was calculated using one-way ANOVA, followed by Holm-Sidak multiple comparisons specifically between three selected groups: AM versus Control, AM-Veh versus AM, AM-Allo versus AM-Veh.

Because aging is associated with decreased levels of hormone, growth, and trophic factors (38, 39), a supplement-reduced media condition was used as an Aging Model (AM) to investigate the effect of Allo on neurons and astrocytes under an aging condition. Neurons (**Figure 1A**) were cultured in Neurobasal media with a reduced amount of B27 (0.2% and 10% Supplement-deprived medium, AM) at day 10 for 4h, then treated with 100 nM Allo (SAFC Inc, UCDQR-001) or vehicle (0.001% EtOH) for 16h in AM medium (40). Astrocytes were cultured in DMEM/F-12 supplemented with 10% charcoal-stripped FBS (Gibco, 12676029, AM) for 24h, then treated with 100 nM Allo or vehicle for 24h in AM medium (41). Allo (100 nM) and the treatment duration were based on previously established analyses across multiple cell types (11, 13, 28). Allo at 100 nM is comparable to physiological pregnancy levels of Allo, and thus, is translationally relevant (42, 43).

#### 2.2.2 Bicuculline and nifedipine treatment

Neurons were cultured in AM medium at day 10 for 4h, then pre-treated with 10 μM bicuculline (GABA_A_ receptor antagonist, Sigma-Aldrich, St. Louis, United States, 14343) in AM medium for 1h, followed by co-incubation of 100 nM Allo and bicuculline in AM medium for 16h. Sixteen-hour nifedipine (Ca^2+^ channel blocker, Sigma-Aldrich, N7634) (28) treatment triggered neuronal cell death in AM medium. Therefore, neurons were cultured in AM medium for 4h, then co-treated with 100 nM Allo and 10 μM nifedipine for 3h.

Astrocytes were cultured in AM medium for 24h, then pre-treated with 10 μM bicuculline or 10 μM nifedipine in AM medium for 1h, followed by co-incubation of 100 nM Allo and either bicuculline or nifedipine in AM medium for 24h.

### 2.3 Seahorse XF-96 Cell Mito Stress Test

Cellular respiratory capacity was determined by XF96e metabolic flux analyzer as previously described (44–46). Hippocampal primary neurons and astrocytes were seeded on poly-D-lysine–coated Seahorse XF96 well plates at a density of 25,000 cells per well and cultured and treated as described above. On the day of assay, cell culture media was changed to unbuffered DMEM medium (pH 7.4, Sigma-Aldrich, D5030) supplemented with 25 mM glucose, 1 mM sodium pyruvate, 31 mM NaCl, and 2 mM glutamine (Gibco, 25030081). Cells were then incubated at 37°C in a non‐CO_2_ incubator for 1h. Oxygen consumption rate (OCR) was used as an indicator of mitochondrial oxidative phosphorylation. Mitochondrial basal respiration, spare and maximal respiratory capacity were determined by sequential acute injection of mitochondrial electron transport chain inhibitors and un-couplers: oligomycin (4 μM, MP Biomedicals, 02151786), FCCP (1 μM, carbonyl cyanide4(trifluoromethoxy)-phenylhydrazone) (TOCRIS Bioscience, 0453), rotenone (1 μM, MP Biomedicals, 02150154), and antimycin (1 μM, Sigma-Aldrich, A-8674). OCR results were normalized to protein concentrations.

### 2.4 Cellular ROS measurement

For neurons, 5 μM CellROX Green Reagent (Invitrogen, C10444) and 10 μg/ml Hoechst-33342 (nuclear counter staining) were added to cell medium. Cells were then incubated for 30 min at 37°C in a humidified 5% CO_2_ incubator, followed by washing 3 times with pre-warmed PBS. The fluorescence intensity was measured using Cytation 5 (BioTek) at 520 nM for CellROX and 461 nm for Hoechst-33342. The individual ROS level was calculated by normalizing CellROX intensity (ROS level) to corresponding Hoechst-33342 intensity (overall cell density).

Astrocytes were incubated with 5 μM CellROX Green Reagent (Invitrogen, C10444) for 30 min at 37°C in a humidified 5% CO_2_ incubator. After three washes with pre-warmed PBS, astrocytes were trypsinized then pelleted by centrifuging and resuspended in 150 μl of PBS with 4′,6-diamidino-2-phenylindole (DAPI). Fluorescence signal was detected by MACSQuant Analyzer Flow Cytometry (Mitenyi Biotec) and analyzed with FlowLogic software.

### 2.5 Immunohistochemistry

Cells were fixed with 4% paraformaldehyde for 15 min and blocked in blocking buffer (5% normal goat serum + 0.3% Triton X-100 in PBS) for 60 min at room temperature. The following primary antibodies were then applied for overnight at 4°C: mouse anti-MAP2 (1:1000, Sigma-Aldrich, M4403), rabbit anti-TOM20 (1:250, Abcam, ab186734), mouse anti-GFAP (1:1000, Chemicon, MAB360), and mouse anti-beta tubulin (1:300, Millipore, MAB1637). Cells were then incubated with Alexa Fluor 488 or 555 goat anti-mouse or anti-rabbit secondary antibodies for 60 min at room temperature. Nuclei were stained with DAPI. Images were captured with Zeiss LSM 880 Airyscan Confocal Microscope or Axiovert 200 M Marianas Digital Fluorescence Microscopy Workstation (Intelligent Imaging Innovations, Denver, CO).

Neurite quantification was conducted using the filament function of Imaris 9.5 software. All analyses were performed with the experimenter blinded to the condition. Fifteen to 20 images were randomly obtained per group and processed with Imaris. The mean neurite count, length, and branch level were calculated per group. The whole procedure was repeated three times, and the mean values from these three independent experiments were plotted and used for statistical analysis.

### 2.6 Mitochondrial morphological subtype analysis

Quantification of mitochondrial morphological subtypes was performed using MitoMorph software (47, 48). Mitochondria were labeled using anti-TOM20 antibody (section 2.5), and individual mitochondria were categorized according to area, morphology, and length into six distinct types: small globules, swollen globules, straight tubules, twisted tubules, branched tubules, and loops. The proportion of fused mitochondria (fusion products) was calculated as the sum of straight tubule, twisting tubule, loop, and branch tubule mitochondrial populations. The numbers of small globules and swollen globules were combined and classified as segregated mitochondria (fission product).

### 2.7 Western blotting

Cells were lysed in M-PER Mammalian Protein Extraction Reagent (Thermo Fisher Scientific, 78501) with 1% Pierce Protease and Phosphatase inhibitor (Thermo Fisher Scientific, A32961). Protein concentrations were then determined by using Protein Assay Dye Reagent (Bio-Rad). Equal amounts of protein (10 μg) were loaded in each well of 12% SDS-PAGE gels (Bio-Rad Laboratories), electrophoresed with a tris/glycine running buffer, and transferred to a polyvinylidine difluoride membrane (0.45 µm PVDF). The following primary antibodies were incubated overnight: rabbit anti-GAP43 (1:1000, Abcam, ab16053), rabbit anti-pERK42/44 (1:1000, Cell Signaling, 4370s), rabbit anti-ERK42/44 (1:1000, Cell Signaling, 4695s), rabbit anti–c-Fos (1:1000, Cell Signaling, 2250), rabbit anti–c-Jun (1:1000, Cell Signaling, 9165), mouse total OXPHOS antibody cocktail (1:1000, Abcam, 110413), rabbit anti-DRP1 (1:1000, Cell Signaling, 8570s), rabbit anti-OPA1 (1:1000, Cell Signaling, 80471s), and mouse anti-Actin (1:10000, Millipore, MAB 1501). HRP-conjugated anti-rabbit or anti-mouse secondary antibody (1:5000, Vector Laboratories, Burlingame, CA) were then applied. The signal was visualized by Pierce SuperSignal Chemiluminescent Substrates or SuperSignal West Pico Chemiluminescent Substrate (Thermo Fisher Scientific, IL, Waltham, MA) and captured by ChemiDoc MP Imaging System (BioRad, Hercules, CA). All band intensities were quantified using Image Lab 6.0.1 (Bio-Rad, Hercules, CA) and normalized to corresponding actin intensity. Relative fold change was calculated in comparison to the AM group.

### 2.8 Mitochondrial DNA copy number measurement

Total DNA was isolated from cells with QIAamp DNA mini kit (Qiagen, Valencia, CA) and analyzed by quantitative PCR. Relative mtDNA/nDNA ratio was calculated as the relative fold change of mt-CO2 (mtDNA) content to β-Globin (nDNA) content. Primers were as follows: mt-CO2 forward: 5′- AAACCAGGTGAACTTCGTCTAT-3′; mt-CO2 reverse: 5′- GGACGTCTTCGGATGAGATTAG -3′; β-Globin forward: 5′- GCTTTCCTGCTCAAATTCCTATC -3′; and β-Globin reverse: 5′- AACACTCCACAGGGCATATC -3′.

### 2.9 Mitochondrial complex IV activity measurement

Mitochondrial complex IV activity was measured by Complex IV Rodent Enzyme Activity Microplate Assay Kit (Abcam, ab109911) following the manufacturer’s instructions. Mitochondrial proteins were extracted from sample homogenates with the concentration of 5.5 mg/ml. Sample (50 μg/200 μl) was added to each well and incubated for 3h at room temperature. The reduced Cytochrome C reagent was then added and the OD550 was measured by Cytation 5 Cell Imaging Reader (BioTek) at 1-min intervals for 2h at 30°C. The initial rate of oxidation of Cytochrome C was calculated within the linear range.

### 2.10 Mitochondrial membrane potential assessment

Mitochondrial membrane potential was assessed by MITO-ID Membrane Potential Detection Kit (Enzo, ENZ-51018-0025) following the manufacturer’s instructions. Cells were washed with 1X assay buffer, then incubated with MITO-ID MP Detection Reagent for 15 min at room temperature. The MMP ratio was detected by Cytation 5 (BioTek). Fluorescence signals were collected at 530/20 nm with 485/20 nm excitation (green) and 570/10 nm with 540/10 nm excitation (orange). The intensity ratio of orange/green was then calculated.

### 2.11 ATP determination

Cellular ATP levels were measured by ATP determination kit (Molecular Probes, A22066) following the manufacturer’s instructions. Luminescence was measured by Cytation 5 (BioTek).

### 2.12 RNA isolation

Cells were lysed in TRIzol® Reagent, followed by chloroform extraction at a volume ratio of 1:5 to that of the TRIzol® Reagent. Ethanol was then used to precipitate nucleic acids from the aqueous phase. RNA was further purified using PureLink™ RNA Mini Kit (Invitrogen, 12183018A) following manufacturer’s instructions. Purelink™ DNase (Invitrogen, 12185010) was used to eliminate DNA contamination. Purified RNA was eluted in RNase-free diH_2_O. RNA concentration and quality were checked by NanoDrop™ One (Thermo Fisher Scientific).

### 2.13 Real-time quantitative PCR

Purified RNA was then reverse transcribed to cDNA using SuperScript VILO Master MIX (Invitrogen, 11755). cDNA (20 ng) was used for rt-PCR with TaqMan Universal PCR Master Mix (Applied Biosystems, 4304437). The following TaqMan primers were used: *Nrf1* (Rn01455958), *Tfam* (Rn00580051), *Ppargc1a* (Rn00580241), and *Actin* (Rn00667869). Target cDNA was amplified and detected using Applied Biosystems QuantStudio 6 Flex system (Thermo Fisher Scientific). Relative gene expression level (fold change) to reference group was calculated by the comparative Ct method.

### 2.14 Gene expression analysis

RNA-seq was conducted at Vanderbilt Technologies for Advanced Genomics (VANTAGE). Only RNA samples with an acceptable RNA quality indicator score (RQI > 7) were used for sequencing. mRNA enrichment and cDNA library preparation were done using a stranded mRNA (poly(A)–selected) sample preparation kit. Sequencing was performed at 150 bp paired-end on NovaSeq6000, targeting 30 million reads per sample. Transcripts were mapped to the rat genome (ensemble release 95) using Salmon 0.14. Tximport V1.6.0 (49) was used to generate a counts table, and DESeq2 V1.18.1 (50) was used to calculate normalized read counts for each gene and/or transcript and to perform expression analysis. Variance stabilizing transformation normalized expression was used for visualization.

### 2.15 Ingenuity pathway analysis

RNA-seq data were processed by the core analysis function of IPA using a *p*-value cutoff of 0.05. The canonical pathways were identified based on enrichment of qualified genes. The upstream regulator analysis predicted activation or inhibition of regulatory molecules based on expression of respective downstream genes and networks compiled from literature and IPA’s Ingenuity knowledge base.

### 2.16 Statistics

Statistical significance was calculated using one-way analysis of variance (ANOVA), followed by Holm-Sidak multiple comparisons specifically between three selected groups: AM versus Control, AM-Veh versus AM, and AM-Allo versus AM-Veh. To assess the effect of pathway inhibitors, a two-way ANOVA was utilized, followed by Holm-Sidak multiple comparisons. Statistical significance and effect size (fold change) for RNA-seq data were calculated by DESeq2 V1.18.1. Comparisons with a *p*-value smaller than 0.05 were considered statistically significant.

### 2.17 Data availability statement

The gene expression data are available through the Gene Expression Omnibus (GEO) repository (GSE229627, https://www.ncbi.nlm.nih.gov/geo/query/acc.cgi?acc=GSE229627).

## 3 Results

### 3.1 Allo promotes neuronal morphological complexity and synaptic plasticity pathways

Morphological complexity of neurons has long been a correlate of information processing and storage capacity (51). Thus, the impact of Allo on morphological indicators of neuronal plasticity was assessed in hippocampal neurons (**Figure 1A**). Relative to control neurons, neurite outgrowth was significantly inhibited in the neurons treated with AM medium (**Figures 1B–E**). The morphological phenotype of AM neurons was characterized by decreased neurite count (*p* < 0.001 vs. Control), neurite length (*p* < 0.001 vs. Control), and neurite branch level (*p* = 0.018 vs. Control). Allo (16h treatment) significantly increased neurite count (*p* = 0.046 vs. AM-Veh) and neurite length (*p* = 0.002 vs. AM-Veh), without affecting neurite branch level. Consistent with the morphological impact, protein level of GAP43 (Growth Associated Protein 43, a marker of synaptic growth) was significantly increased in Allo-treated neurons (**Figure 1F**, *p* = 0.028 vs. AM-Veh). No differences in neurite growth were observed between AM and AM-Veh groups, confirming the specificity of the Allo effect.

**Figure 2:**
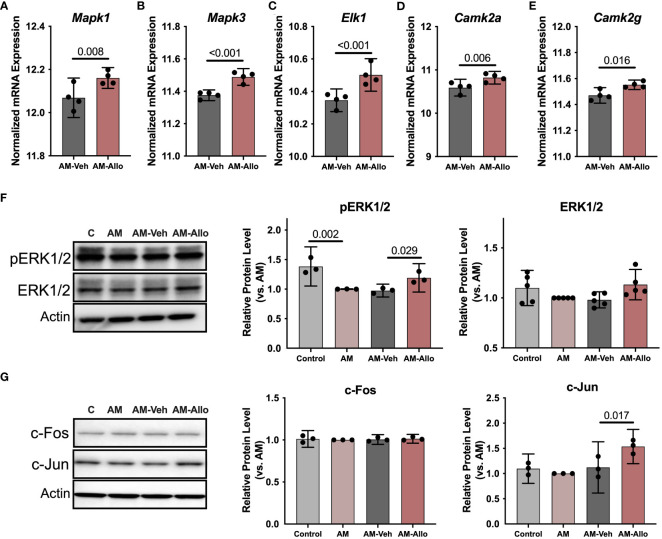
Allo activated Ca^2+^ signaling cascades associated with neuroplasticity in hippocampal neurons. Allo induced significant increases in gene expression levels of *Mapk1* **(A)**, *Mapk3* **(B)**, *Elk1* **(C)**, *Camk2a* **(D)** and *Camk2g* **(E)** (*n* = 4). **(F)** Allo treatment significantly restored protein levels of pERK1/2 (*n* = 3, one-way analysis of variance (ANOVA), F (3, 8) = 14.95, *p* = 0.001, η^2^ = 0.849) and induced a trend toward increased ERK1/2 protein levels (*n* = 5, one-way ANOVA, F (3, 16) = 2.847, *p* = 0.071, η^2^ = 0.348). **(G)** Protein levels of c-Jun were significantly increased by Allo treatment (*n* = 3, one-way ANOVA, F (3, 8) = 9.113, *p* = 0.006, η^2^ = 0.774). All bar graphs are presented as mean ± 95% CI with individual data points. Statistical significance for RNA-seq data was calculated by DESeq2 V1.18.1. Other data were analyzed using one-way ANOVA, followed by Holm-Sidak multiple comparisons specifically between three selected groups: AM versus Control, AM-Veh versus AM, AM-Allo versus AM-Veh.

To investigate the impact of Allo on transcriptional pathways relevant to neuroplasticity and to identify mechanistic pathways, unbiased exploratory transcriptomic analysis was conducted in Veh and Allo-treated (16h treatment) hippocampal neurons in parallel to morphological analyses. As shown in **Table 1**, transcriptomic analysis revealed that Allo significantly upregulated gene expression associated with synaptogenesis and synaptic long-term potentiation, which is consistent with neuroplasticity. Mechanistically, Ca^2+^ and CREB (cAMP-responsive element-binding protein) signaling were identified as the top pathways activated by Allo (**Table 1**), which replicated our previous findings (28). Transcriptomic profiling indicated significant increases in the gene expression of key activators of Ca^2+^-CREB signaling, including *Mapk1* (Mitogen-activated protein kinase 1, ERK2, **Figure 2A**, *p* = 0.008, fold change = 1.074), *Mapk3* (Mitogen-activated protein kinase 3, ERK1, **Figure 2B**, *p* < 0.001, fold change = 1.101), *Elk1* (ETS transcription factor ELK1, **Figure 2C**, *p* < 0.001, fold change = 1.181), *Camk2a* (Calcium/Calmodulin dependent protein kinase II Alpha, **Figure 2D**, *p* = 0.006, fold change = 1.250), and *Camk2g* (Calcium/Calmodulin dependent protein kinase II gamma, **Figure 2E**, *p* = 0.016, fold change = 1.072). Consistent with transcriptional analyses, Allo (16h treatment) significantly increased protein levels of pERK1/2 (**Figure 2F**, *p* = 0.029 vs. AM-Veh) and induced a trend toward increased ERK1/2 protein levels (*p* = 0.077 vs. AM-Veh). Further, protein level of c-Jun, another effector of Ca^2+^ signaling, was also significantly increased by Allo treatment (16h treatment, **Figure 2G**, *p* = 0.017 vs. AM-Veh).

**Table 1:** Allo-activated neuroplasticity and calcium signaling pathways.

Pathway	*Z*-score	*P*-value	Significantly changed genes (Red indicates upregulation, green indicates downregulaton)
Synaptogenesis pathway	4.111	1.57E-12	*Adcy1, Adcy5, Afdn, Arhgef7, Bdnf, Cadm1, Camk2a, Camk2g, Cdh20, Clasp2, Cntnap1, Efnb3, Epha4, Fyn, Grin3a, Grm1, Grm3, Grm4, Grm5, Lrp1, Lrp8, Mapk1, Mapk3, Mapt, Nrxn1, Nrxn2, Pik3cd, Pik3r1, Prkacb, Rap1b, Rasd2, Reln, Rhoa, Shc3, Snca, Stx1b, Syn2, Syn3, Syt4, Syt7, Syt13*
Synaptic long-term potentiation	2.982	6.54E-08	*Adcy1, Camk2a, Camk2g, Grin3a, Grm1, Grm3, Grm4, Grm5, Itpr2, Mapk1, Mapk3, Plch2, Ppp1r1a, Ppp3ca, Prkacb, Prkcg, Prkcz, Rap1b, Rasd2, Rps6ka1*
Ca2+ signaling	3.441	5.90E-06	*Atp2b3, Cacna1e, Cacna2d3, Camk1d, Camk2a, Camk2g, Camkk1, Chrna7, Grin3a, Hdac9, Itpr2, Mapk1, Mapk3, Mef2c, Myh11, Myl9, Ppp3ca, Prkacb, Rap1b, Rcan2, Ryr2, Ryr3, Slc8a2*
CREB signaling	3.202	7.31E-08	*Adcy1, Adcy5, Adgrg6, Adgrl1, Adgrv1, Bdkrb2, Bmp6, Cacna1e, Cacna2d3, Camk2a, Camk2g, Cnr1, Elk1, F2r, Fgfr3, Fzd7, Gnao1, Gnb3, Gng2, Gng3, Gng4, Gng5, Gpr17, Gpr62, Gpr85, Gpr139, Gpr155, Grm1, Grm3, Grm4, Grm5, Hrh3, Htr1a, Itpr2, Mapk1, Mapk3, Ntsr1, Pik3cd, Pik3r1, Plch2, Prkacb, Prkcg, Prkcz, Rap1b, Rasd2, Rps6ka1, S1pr3, Shc3, Tgfb2, Tgfbr3*

Collectively, these data indicate that Allo-induced neuroplasticity was associated with activation of the Ca^2+^/CREB signaling pathway.

### 3.2 Allo promotes neuronal mitochondrial function

**Figure 3:**
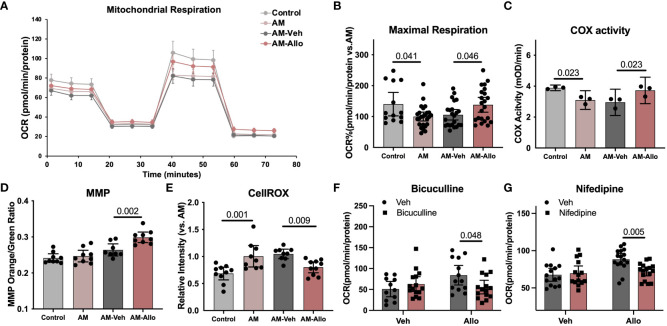
Allo restored mitochondrial respiration and oxidative phosphorylation via GABA_A_ and L-type calcium channel activation while preventing free radical generation in hippocampal neurons. **(A)** Representative mitochondrial stress test results using the Seahorse XF96 Extracellular Flux Analyzer (mean ± S.E.M.). **(B)** Allo treatment restored AM-induced decrease in maximal mitochondrial respiration. (*n* = 13–25 wells per group, one-way analysis of variance (ANOVA), F (3, 79) = 4.096, *p* = 0.009, η^2^ = 0.135). **(C)** AM-impaired COX activity was restored by Allo treatment (*n* = 3, one-way ANOVA, F (3, 8) = 8.386, *p* = 0.008, η^2^ = 0.759). **(D)** Allo treatment significantly increased the ratio of mitochondria with high MMP (*n* = 8–9, one-way ANOVA, F (3, 31) = 16.78, *p* < 0.001, η^2^ = 0.619). **(E)** Allo treatment significantly reversed AM-induced increase in CellROX levels (*n* = 9–10, one-way ANOVA, F (3, 34) = 8.973, *p* < 0.001, η^2^ = 0.442). **(F)** Bicuculline blocked the effect of Allo on promoting mitochondrial maximal respiration (*n* = 11–15 wells per group, two-way ANOVA, interaction: F (1, 50) = 5.213, *p* = 0.027, 9.003% of total variation; Allo: F (1, 50) = 2.198, *p* = 0.145, 3.795% of total variation; bicuculline: F (1, 50) = 0.8641, *p* = 0.357, 1.492% of total variation). **(G)** Nifedipine blocked the effect of Allo on promoting mitochondrial maximal respiration (*n* = 14–16 wells per group, two-way ANOVA, interaction: F (1, 56) = 5.924, *p* = 0.018, 7.808% of total variation; Allo: F (1, 56) = 9.577, *p* = 0.003, 12.62% of total variation; nifedipine: F (1, 56) = 3.704, *p* = 0.059, 4.882% of total variation). All bar graphs are presented as mean ± 95% CI with individual data points. Statistical significance was calculated using one-way ANOVA, followed by Holm-Sidak multiple comparisons specifically between three selected groups: AM versus Control, AM-Veh versus AM, and AM-Allo versus AM-Veh. The inhibitor’s effect was analyzed by two-way ANOVA, followed by Holm-Sidak multiple comparisons.

Our previous *in-vivo* analyses indicated that Allo promoted brain mitochondrial respiration (10). Herein, we investigated the cellular contribution of the Allo-induced increase in mitochondrial respiration from neurons. Neuronal mitochondrial maximal respiration (**Figures 3A, B**, *p* = 0.041 vs. Control) was reduced in the AM group. In contrast, Allo treatment (16h treatment) significantly increased mitochondrial maximal respiration (**Figures 3A, B**, *p* = 0.046 vs. AM-Veh). Furthermore, Allo treatment reversed the AM-induced decrease in Complex IV (COX) activity (**Figure 3C**, *p* = 0.023 vs. AM-Veh). A significant increase in the ratio of high membrane potential mitochondria was also observed in Allo-treated group compared to Veh-treated group (**Figure 3D**, *p* = 0.002 vs. AM-Veh), indicative of improved mitochondrial efficiency. Furthermore, Allo (16h treatment) significantly decreased ROS levels (*p* = 0.009 vs. AM-Veh) relative to the rise in cellular oxidative status observed in the AM group (**Figure 3E**, *p* = 0.001 vs. Control). Consistent with the transcriptomic profiles, inhibiting the GABA_A_ receptor with bicuculline (**Figure 3F**, *p* = 0.048 vs. Allo) or inhibiting the L-type Ca^2+^ channel with nifedipine (**Figure 3G**, *p* = 0.005 vs. Allo) significantly blocked this Allo-induced increase of mitochondrial maximal respiration.

Collectively, the data indicate that Allo significantly increased neuronal mitochondrial respiration and oxidative phosphorylation while preventing free radical generation via GABA_A_ and L-type Ca^2+^ channel-dependent Ca^2+^ signaling activation in hippocampal neurons.

### 3.3 In hippocampal neurons, Allo does not affect mitochondrial biogenesis

**Figure 4:**
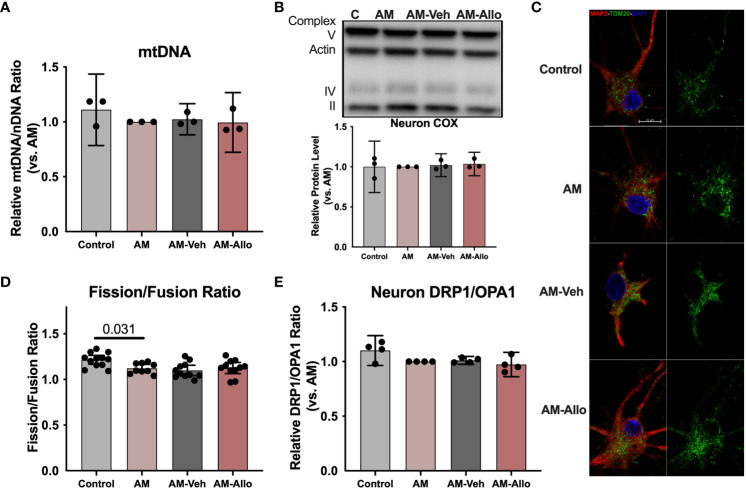
In hippocampal neurons, Allo does not affect mitochondrial biogenesis or dynamics. **(A)** Mitochondrial DNA copy number remained unchanged following Allo treatment (*n* = 3). **(B)** Mitochondrial complex IV protein levels were not changed by Allo treatment (*n* = 3). **(C)** Representative neuron images labeled using anti-MAP2 and anti-TOM20 antibodies and DAPI. **(D)** The overall mitochondrial fission/fusion ratio was significantly reduced in the AM group (Mitochondria were labeled using the anti-TOM20 antibody, *n* = 9–12, one-way ANOVA, F (3, 39) = 4.848, *p* = 0.006, η^2^ = 0.272) and was not reversed by Allo treatment. **(E)** The DRP1/OPA1 protein ratio (*n* = 4) was not affected by Allo treatment. All bar graphs are presented as mean ± 95% CI with individual data points. Statistical significance was calculated using one-way ANOVA, followed by Holm-Sidak multiple comparisons specifically between three selected groups: AM versus Control, AM-Veh versus AM, and AM-Allo versus AM-Veh.

Improved mitochondrial function could be coupled with mitochondrial biogenesis and dynamic restructuring (52). To assess whether the observed Allo-induced rise in neuronal mitochondrial function (section 3.2 above) required mitochondrial biogenesis or dynamics, Allo regulation of mitochondrial DNA copy number, complex protein levels, and mitochondrial subtype distribution was determined (16h treatment). In hippocampal neurons, Allo did not impact mitochondrial DNA copy number (**Figure 4A**). Consistent with this outcome, mitochondrial complex IV (COX) protein levels remained unchanged by Allo treatment (**Figure 4B**). AM significantly reduced mitochondrial fission/fusion ratio (**Figures 4C, D**, *p* = 0.031 vs. Control), without changing the protein expression ratio of DRP1 (Dynamin-1-like protein, an essential protein for mitochondrial fission) to OPA1 (OPA1 mitochondrial dynamin like GTPase, an essential protein for mitochondrial fusion) (**Figure 4E**). Allo did not affect the overall mitochondrial fission/fusion ratio (**Figures 4C, D**) nor the balance between DRP1 and OPA1 (**Figure 4E**). Together, Allo-induced improvement of mitochondrial function was not mediated via the direct regulation of mitochondrial biogenesis or dynamics but rather through promoting COX activity and increasing the ratio of high membrane potential mitochondria that promote mitochondrial efficiency (section 3.2 above).

### 3.4 Allo decreased oxidative stress, inhibited apoptosis, and decreased pro-inflammation activation in astrocytes

**Figure 5:**
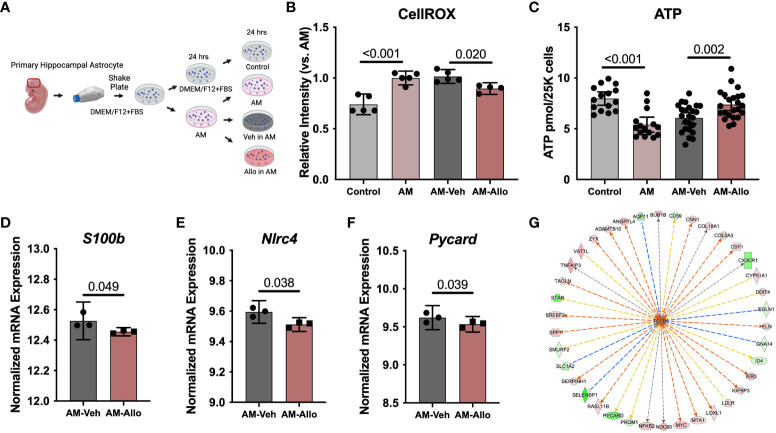
Allo decreased AM-induced oxidative stress and pro-inflammatory signaling in astrocytes. **(A)** The schematic diagram of Allo treatment (Created with BioRender.com). **(B)** Allo treatment significantly reversed AM-induced increase in CellROX levels (*n* = 4–5, one-way ANOVA, F (3, 15) = 22.03, *p* < 0.001, η^2^ = 0.815). **(C)** ATP levels were significantly restored by Allo treatment (*n* = 15–24, one-way ANOVA, F (3, 73) = 13.25, *p* < 0.001, η^2^ = 0.353). Relative to AM-Veh group, Allo treatment decreased the RNA levels of *S100b* **(D)** (*n* = 3), *Nlrc4* **(E)** (*n* = 3) and *Pycard* **(F)** (*n* = 3). **(G)** IPA predicted TGFB1 would be activated by Allo treatment. All bar graphs are presented as mean ± 95% CI with individual data points. Statistical significance for RNA-seq data was calculated by DESeq2 V1.18.1. Other data were analyzed using one-way ANOVA, followed by Holm-Sidak multiple comparisons specifically between three selected groups: AM versus Control, AM-Veh versus AM, and AM-Allo versus AM-Veh.

Astrocyte-related neuroinflammation and oxidative stress are observed in and can contribute to Alzheimer’s disease (AD) pathology (53, 54). Therefore, the effect of Allo (24h treatment, **Figure 5A**) on astrocytic oxidative stress status and inflammatory activation was investigated. As shown in **Figure 5B**, AM induced an increase in cellular ROS levels (*p* < 0.001 vs. Control). Allo significantly decreased ROS levels (*p* = 0.020 vs. AM-Veh), which was accompanied by restored cellular ATP levels (**Figure 5C**, *p* = 0.002 vs. AM-Veh). These results indicated that Allo relieved astrocytic oxidative stress and promoted ATP generation. Consistent with these findings, broader transcriptomic analyses revealed that Allo treatment significantly activated stress response EIF2 (eukaryotic initiation factor-2) signaling and inhibited apoptosis signaling (**Table 2**). Notably, the inflammasome signaling pathway was inhibited by Allo treatment. Relative to Veh-treated group, a significant decrease in gene expression was detected for *S100b* (**Figure 5D**, *p* = 0.049, fold change = 0.940), *Nlrc4* (NLR family CARD domain-containing protein 4, **Figure 5E**, *p* = 0.038, fold change = 0.614) and *Pycard* (PYD and CARD domain containing, **Figure 5F**, *p* = 0.039, fold change = 0.615). In addition, IPA upstream regulator analysis predicted that Allo treatment would activate TGFB1 (Transforming growth factor b1), a key factor in preventing inflammation in neurological disorders (**Figure 5G**) (55). Together, these results indicated that Allo prevented astrocytic oxidative stress and inhibited apoptosis and pro-inflammation activation.

**Table 2:** Allo significantly increased stress response EIF2 signaling and inhibited apoptosis signaling in astrocytes.

Pathway	*Z*-score	*P*-value	Significantly changed genes (red indicates upregulation, green indicates downregulation)
EIF2 signaling	0.905	1.12E-09	*Eif3m, Rps13, Myc, Rap2b, Rpl12*(LOC102555453)*, Rpl15, Rpl21, Rpl28, Rpl30*(LOC100362027)*, Rpl36, Rpl13a, Rpl37a, Rplp1, Rps10l1, Rps16, Rps21, Rps23, Rps28, Rps29, Rps27l, Rps3a*(LOC100365839)
Apoptosis signaling	−0.447	4.11E-02	*Cycs, Lmna, Nfkb2, Plcg1, Rap2b*

### 3.5 Allo restored astrocytic mitochondrial function

**Figure 6:**
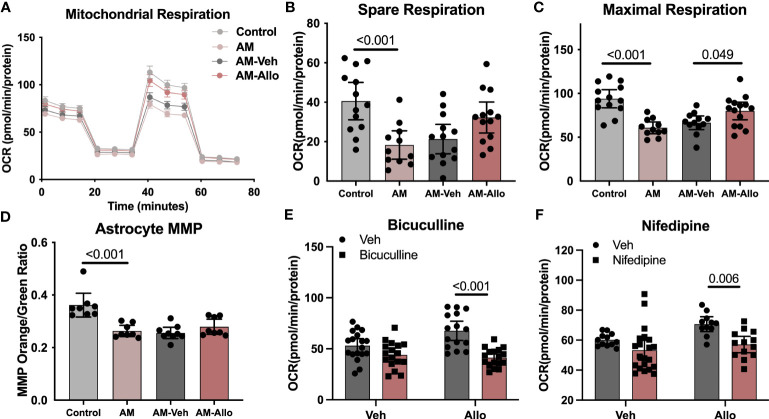
Allo restored astrocytic mitochondrial function via GABA_A_ and L-type calcium channels. **(A)** Representative mitochondrial stress test results using the Seahorse XF96 Extracellular Flux Analyzer (mean ± S.E.M.). AM significantly reduced astrocytic mitochondrial spare **(B)** (One-way analysis of variance (ANOVA), F (3, 46) = 7.483, *p* < 0.001, η^2^ = 0.328) and maximal respiration **(C)** (one-way ANOVA, F (3, 46) = 12.26, *p* < 0.001, η^2^ = 0.444). Allo treatment induced a trend toward increased spare respiration and significantly increased maximal respiration **(B, C)** (*n* = 11–14 wells per group). **(D)** The AM-induced significant decrease in astrocytic mitochondrial membrane potential (*n* = 8–9, one-way ANOVA, F (3, 29) = 14.03, *p* < 0.001, η^2^ = 0.592) was not reversed by Allo treatment. **(E)** Bicuculline blocked Allo’s effect on promoting mitochondrial maximal respiration (*n* = 15–18 wells per group, two-way ANOVA, interaction: F (1, 63) = 7.005, *p* = 0.010, 7.05% of total variation; Allo: F (1, 63) = 3.256, *p* = 0.076, 3.277% of total variation; bicuculline: F (1, 63) = 28.29, *p* < 0.001, 28.47% of total variation). **(F)** Nifedipine blocked Allo’s effect on promoting mitochondrial maximal respiration (*n* = 11–21 wells per group, two-way ANOVA, interaction: F (1, 53) = 1.340, *p* = 0.252, 1.834% of total variation; Allo: F (1, 53) = 6.115, *p* = 0.017, 8.367% of total variation; nifedipine: F (1, 53) = 12.53, *p* < 0.001, 17.14% of total variation). All bar graphs are presented as mean ± 95% CI with individual data points. Statistical significance was calculated using one-way ANOVA, followed by Holm-Sidak multiple comparisons specifically between three selected groups: AM versus Control, AM-Veh versus AM, and AM-Allo versus AM-Veh. The inhibitor’s effect was analyzed by two-way ANOVA, followed by Holm-Sidak multiple comparisons.

Allo-induced reduction in oxidative stress and increased ATP production is consistent with improved mitochondrial function. Furthermore, because the inflammasome can be activated by ROS-generating mitochondria (56), enhanced mitochondria function would lead to inhibition of inflammasome activation. Therefore, we hypothesized that the effect of Allo to reverse astrocytic oxidative stress, apoptosis, and pro-inflammation activation was associated with its regulation of mitochondrial function. To address this hypothesis, mitochondrial respiration and membrane potential were assessed. AM significantly reduced astrocytic mitochondrial spare (**Figures 6A, B**, *p* < 0.001 vs. Control) and maximal respiration (**Figures 6A, C**, *p* < 0.001 vs. Control), which was accompanied by a significant decrease in the ratio of mitochondria with high membrane potential (**Figure 6D**, *p* < 0.001 vs. Control). Allo treatment (24h treatment) induced a trend toward increased spare respiration (**Figure 6B**, *p* = 0.077 vs. AM-Veh) and increased maximal respiration significantly (**Figure 6C**, *p* = 0.049 vs. AM-Veh), without affecting mitochondrial membrane potential (**Figure 6D**). Furthermore, bicuculline (**Figure 6E**, *p* < 0.001 vs. Allo) and nifedipine (**Figure 6F**, *p* = 0.006 vs. Allo) significantly blocked the Allo effect on promoting mitochondrial maximal respiration. Together, these results indicate that Allo restored astrocytic mitochondrial function, which required both GABA_A_ and L-type Ca^2+^ channel activation.

### 3.6 Allo promoted astrocytic mitochondrial biogenesis and dynamics

**Figure 7:**
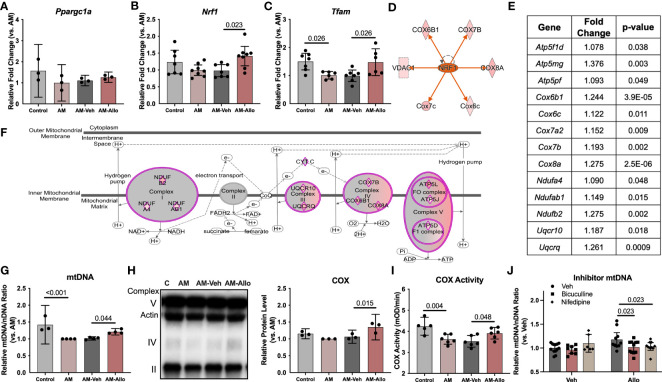
Allo promoted astrocytic mitochondrial biogenesis via NRF1-TFAM signaling. **(A)** *Ppargc1a* (PGC1alpha) mRNA level was not affected by Allo treatment (*n* = 3). Allo treatment significantly increased *Nrf1* **(B)** (*n* = 7–8, one-way ANOVA, F (3, 26) = 3.985, *p* = 0.018, η^2^ = 0.315) and *Tfam* **(C)** (*n* = 6–7, one-way ANOVA, F (3, 22) = 5.577, *p* = 0.005, η^2^ = 0.432) mRNA levels compared to AM-Veh group. **(D)** Upstream regulator analysis predicted NRF1 would be activated by Allo treatment. **(E, F)**. Allo significantly increased nuclear-encoded gene expression for mitochondrial complex I, III, IV, and V (in purple diamonds, IPA). **(G)** Allo treatment significantly increased mitochondrial DNA copy number (*n* = 3–4, one-way ANOVA, F (3, 11) = 12.13, *p* < 0.001, η^2^ = 0.768). Allo treatment specifically and significantly increased COX protein levels **(H)**, (*n* = 3, one-way ANOVA, F (3, 8) = 8.328, *p* = 0.008, η^2^ = 0.758) which was coupled with a significant increase in COX activity **(I)** (*n* = 5–6, one-way ANOVA, F (3, 19) = 7.539, *p* = 0.002, η^2^ = 0.544). **(J)** Bicuculline and nifedipine significantly inhibited Allo effect on promoting mitochondrial biogenesis (*n* = 6–13, two-way ANOVA, interaction: F (2, 52) = 3.540, *p* = 0.036, 10.64% of total variation; Allo: F (1, 52) = 2.112, *p* = 0.152, 3.174% of total variation; inhibitor: F (2, 52) = 2.630, *p* = 0.082, 7.906% of total variation). All bar graphs are presented as mean ± 95% CI with individual data points. Statistical significance was calculated using one-way ANOVA, followed by Holm-Sidak multiple comparisons specifically between three selected groups: AM versus Control, AM-Veh versus AM, and AM-Allo versus AM-Veh. The inhibitor’s effect was analyzed by two-way ANOVA, followed by Holm-Sidak multiple comparisons.

To investigate whether the Allo-induced increase in astrocytic mitochondrial function was associated with regulation of mitochondrial biogenesis or dynamics, quantitative transcriptomic analyses of key mitochondrial biogenesis regulators, mitochondria DNA copy number, and protein levels were conducted (24h treatment). Real-time PCR outcomes indicated that Allo significantly increased both *Nrf1* (nuclear respiratory factor 1, **Figure 7B**, *p* = 0.023 vs. AM-Veh) and *Tfam* (mitochondrial transcription factor A, **Figure 7C**, *p* = 0.026 vs. AM-Veh) mRNA levels without impacting *Ppargc1a* (PGC1α, PPARG coactivator 1 alpha, **Figure 7A**) mRNA expression. Consistent with transcriptional data, upstream regulator pathway analysis predicted that Allo treatment would activate NRF1 signaling (**Figure 7D**). Allo treatment significantly increased OXPHOS gene expression (**Figures 7E, F**), including *Ndufa4*, *Ndufab1*, *Ndufb2*, *Uqcr10*, *Uqcrq*, *Cox6b1*, *Cox6c*, *Cox7a2*, *Cox7b*, *Cox8a*, *Atp5f1d*, *Atp5mg*, and *Atp5pf*, and significantly increased astrocytic mitochondrial DNA copy number (**Figure 7G**, *p* = 0.044 vs. AM-Veh). Consistent with the transcriptomic profile, Allo significantly increased mitochondrial COX protein level (**Figure 7H**, *p* = 0.015 vs. AM-Veh) and activity (**Figure 7I**, *p* = 0.048 vs. AM-Veh). Inhibiting either the GABA_A_ receptor with bicuculline or the L-type Ca^2+^ channel with nifedipine (**Figure 7J**, *p* = 0.023 and *p* = 0.023 vs. Allo, respectively) blocked the Allo-induced increase in mitochondrial DNA copy number. These data indicated that Allo regulation of mitochondrial biogenesis required both GABA_A_ and L-type Ca^2+^ channel activation.

**Figure 8:**
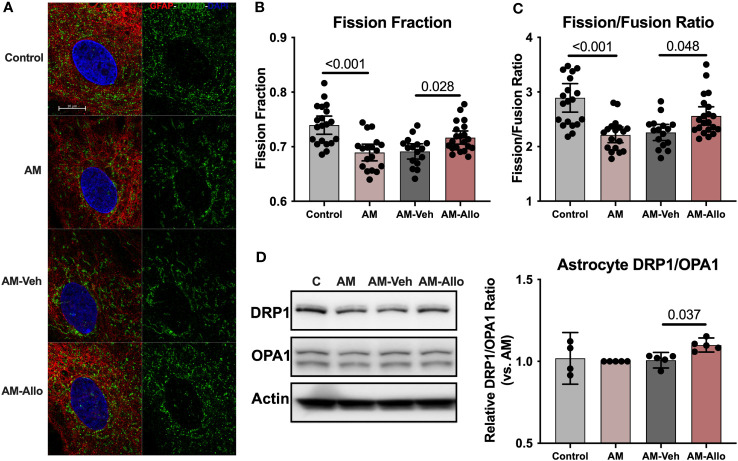
Allo relieved AM-induced astrocytic mitochondrial hyperfusion by increasing DRP1/OPA1 protein ratio. **(A)** Representative astrocyte images labeled with anti-GFAP and anti-TOM20 antibodies and DAPI. Allo treatment significantly increased mitochondrial fission product fraction **(B)** (Mitochondria were labeled using the anti-TOM20 antibody, one-way ANOVA, F (3, 71) = 11.56, *p* < 0.001, η^2^ = 0.328) and restored the mitochondrial fission/fusion ratio compared to AM-Veh groups **(C)**, (*n* = 16–21, one-way ANOVA, F (3, 71) = 11.79, *p* < 0.001, η^2^ = 0.333). **(D)** The DRP1/OPA1 protein ratio was significantly increased by Allo treatment (*n* = 4–5, one-way ANOVA, F (3, 15) = 3.976, *p* = 0.029, η^2^ = 0.443). All bar graphs are presented as mean ± 95% CI with individual data points. Statistical significance was calculated using one-way ANOVA, followed by Holm-Sidak multiple comparisons specifically between three selected groups: AM versus Control, AM-Veh versus AM, and AM-Allo versus AM-Veh.

At the structural level, AM induced hyperfused mitochondrial networks in astrocytes as indicated by a significantly decreased mitochondrial fission fraction (*p* < 0.001 vs. Control) and fission/fusion ratio (*p* < 0.001 vs. Control) (**Figures 8A–C**). Allo treatment (24h treatment) significantly relieved mitochondrial hyperfusion (**Figure 8A**) by increasing the fraction of mitochondrial fission product (**Figure 8B**, *p* = 0.028 vs. AM-Veh), which led to the corresponding restoration of the fission/fusion ratio in the Allo-treated group (**Figure 8C**, *p* = 0.048 vs. AM-Veh). Consistent with the mitochondrial restructuring, the protein expression ratio of DRP1 to OPA1 was also significantly increased by Allo treatment (**Figure 8D**, *p* = 0.037 vs. AM-Veh).

Collectively, the data indicate that Allo promoted astrocytic mitochondrial biogenesis through NRF1-TFAM activation and increased DRP1/OPA1 protein ratio to reverse stress-induced hyperfused mitochondrial network to restore mitochondrial and bioenergetic homeostasis (57).

## 4 Discussion

Our previous analyses demonstrated that Allo exerted pleiotropic actions in the brain that included neurogenesis, oligogenesis, human and rodent neural stem cell regeneration, improved cognitive function, enhanced glucose metabolism, increased mitochondrial respiration and biogenesis, and reduced burden of AD pathology (1–3, 9, 10, 21, 26, 58). Herein, we investigated the potential of a common mechanistic signaling pathway that could serve to unify the pleiotropic outcomes induced by Allo in neural cells. Outcomes of those analyses provide strong support that Allo’s pleiotropic actions activate and are dependent upon Ca^2+^ signaling. Allo activation of Ca^2+^ signaling in both neurons and astrocytes serves to integrate multiple systems of biology critical to sustained brain function and health.

### 4.1 Ca^2+^ signaling as a common mechanistic pathway of Allo pleiotropic action in brain

**Figure 9:**
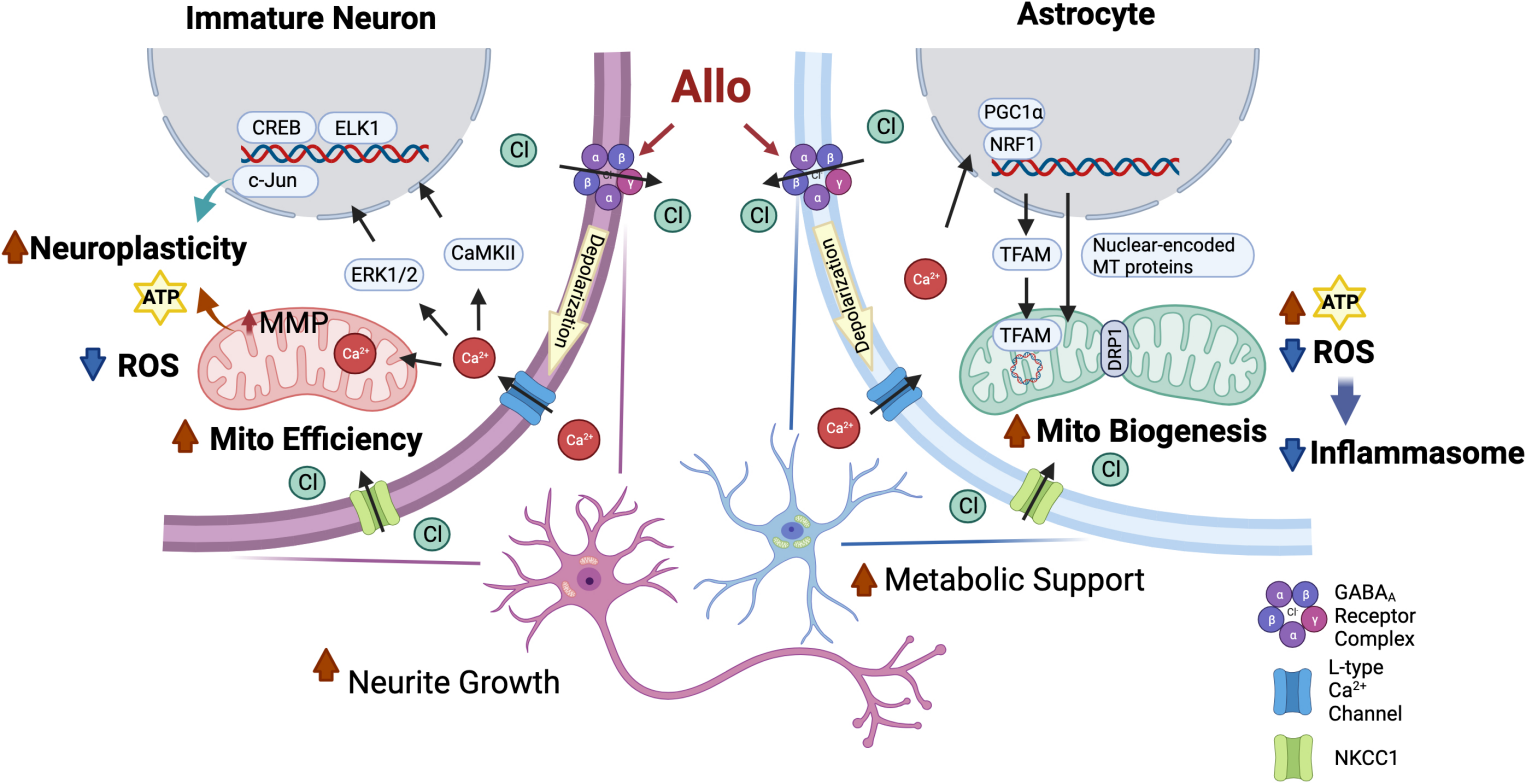
Common mechanisms of action of Allo to activate neuronal and astrocytic functions required for brain regeneration. Both hippocampal immature neurons and astrocytes exhibit a high-intracellular chloride concentration (31, 34). Allo activation of GABA_A_ channels results in Cl^-^ efflux and subsequent depolarization of the membrane potential and activation of the voltage-dependent L-type Ca^2+^ channel, which in turn results in an influx of Ca^2+^ (3, 35). In immature neurons, increased intracellular Ca^2+^ levels activate downstream CREB/ELK1/c-Jun signaling through CaMKII and ERK1/2 activation, which in turn lead to increased neuroplasticity and reduced oxidative stress. The rise in intracellular Ca^2+^ results in sequestration of Ca^2+^ into the mitochondria that promotes mitochondrial respiration and mitochondrial membrane potential. In astrocytes, GABA_A_ and L-type calcium channel activation triggers the activation of NRF1-TFAM signaling and increases the DRP1/OPA1 protein ratio to increase mitochondrial biogenesis and dynamics and consequent ATP production, potentially via calcium-induced PGC-1α activation (59, 60). Furthermore, Allo reduced oxidative stress, thereby reducing activation of inflammasome and apoptosis signaling.

Key to calcium signaling as a unifying mechanism of Allo action is the expression of the NKCC1 chloride transporter (**Figure 9**, created with BioRender.com). Hippocampal immature neurons and astrocytes express NKCC1 and consequently harbor a high-intracellular chloride concentration. In NKCC1-expressing immature neurons, Allo activation of GABA_A_ channels results in Cl^−^ efflux and subsequent depolarization of the membrane potential and activation of the voltage-dependent L-type Ca^2+^ channel, which in turn results in an influx of Ca^2+^ (3). The Allo-induced rise in intracellular Ca^2+^ activates CREB/ELK1/c-Jun signaling through CaMKII and ERK1/2 activation. In neurons, activation of CREB/ELK1/c-Jun signaling is a well-established neural plasticity pathway (61, 62), which is consistent with the significant increases in both neuronal morphology and activation of transcriptional networks required for synaptic plasticity. The rise in intracellular Ca^2+^ results in sequestration of Ca^2+^ into the mitochondria, increasing mitochondrial membrane potential and promoting mitochondrial respiration (63).

Astrocytes also express both GABA_A_ receptors and NKCC1 transporters and produce and release GABA (64). Allo activation of GABA_A_ receptors in astrocytes results in activation of the voltage-dependent L-type Ca^2+^ channel (35) and consequent activation of NRF1-TFAM signaling and increased DRP1/OPA1 protein ratio, promoting mitochondrial biogenesis and dynamics, potentially via Ca^2+^-induced PGC-1α activation (59, 60). Furthermore, Allo reduced oxidative stress and enhanced mitochondrial function, leading to increased ATP concentration and reduced oxidative stress, thereby reducing activation of inflammasome and apoptosis signaling.

These results were consistent with our previous findings demonstrating that Allo induced a rapid and transient increase in cytosolic Ca^2+^ by GABA_A_ and L-type calcium channel activation in embryonic hippocampal neurons from days *in vitro* 3–10 (28) and increased phosphorylation of CREB *in vivo* (65).

### 4.2 Allo regulation of mitochondrial function

The quality and capacity of mitochondria decline with aging, and this is linked to the development of age-related diseases (66). To maintain an adequate and functional mitochondrial population, mitochondrial biogenesis and dynamics are highly regulated. Herein, our results demonstrated that *in-vitro* Allo treatment promoted mitochondrial function in hippocampal neurons through increased mitochondrial efficiency whereas in astrocytes Allo promoted mitochondrial biogenesis and dynamics.

Allo regulation of mitochondria in hippocampal neurons targeted mitochondrial respiratory efficiency without affecting mitochondrial number, potentially through direct stimulation of mitochondrial oxidative phosphorylation due to increased sequestration of Ca^2+^ into the mitochondria (63). Although mitochondrial dynamics overall were not affected by Allo, the restoration of neurite growth in neurons upon Allo treatment suggested improved axonal transport of mitochondria, which is required for synaptic plasticity (67). In astrocytes, Allo activated mitochondrial biogenesis through the NRF1-TFAM signaling pathway and attenuated the fused mitochondrial phenotype by upregulating DRP1/OPA1 protein ratio.

Consistent with our previous *in-vivo* findings that Allo improved mitochondrial function and reduced expression of mitochondrial fusion-related genes in ovariectomized 3xTgAD females (10), *in-vitro* results reported herein indicate that Allo promotes mitochondrial function in both immature neurons and astrocytes through cell-specific mechanisms. Thus, Allo-mediated potentiation of mitochondrial function observed *in vivo* is likely the result of promoting mitochondrial respiration in immature neurons and mitochondrial biogenesis in astrocytes.

### 4.3 Mechanisms underlying pleiotropic action of Allo in two cell types

Transcriptomic pathway analysis revealed that, in hippocampal immature neurons, Allo activated the Ca^2+^/CREB signaling pathway which resulted in the activation of synaptogenesis and long-term potentiation pathways. Furthermore, a rise in mitochondrial Ca^2+^ via activation of L-type calcium channels stimulates mitochondrial oxidative respiration, which is essential for induction and maintenance of neuroplasticity (68). This Allo-induced calcium signaling depends on a high-intracellular chloride concentration, making it mechanistically inapplicable to mature neurons. However, our previous analyses demonstrated that Allo promotes neurogenesis in the 3xTgAD and normal-aged mouse hippocampus (13, 21, 26, 28). Furthermore, Allo promotes neuronal and oligodendrocyte differentiation of neural stem cells (11). These findings collectively support the potential of Allo to promote regeneration in the developing, adult and AD.

In astrocytes, Allo promoted mitochondrial biogenesis that increased mitochondrial respiratory capacity, which was dependent upon L-type calcium channel activation. Furthermore, Allo significantly reduced inflammasome activation by downregulating the expression of *Nlrc4* and *Pycard*. Additionally, Allo activated the TGFB1 signaling pathway, consistent with Allo’s effect on TGFB1 signaling in cancer cell models (69). NLRC4 has been reported to mediate inflammasome activation in microglia and astrocytes (70), which play an important role in neuroinflammation associated with AD (71). Furthermore, TGFB1 has been reported to exert neuroprotective effects in AD, and its deficiency is associated with Aβ pathology and neurofibrillary tangle formation (72). Therefore, Allo’s anti-inflammation effect, manifested as inhibition of NLRC4-mediated inflammasome activation and activation of TGFB1 pathway, could contribute to its therapeutic effect in reducing Aβ burden observed *in vivo* (21).

In conclusion, Allo is a neurosteroid with pleiotropic actions in the brain that span neural regeneration, bioenergetics, and immune systems of biology, which collectively impact synaptic plasticity, cognition, burden of neurodegenerative disease, and recovery of brain function. Unlike classic steroids, Allo exerts pleiotropic effects through non-nuclear receptors. Outcomes of analyses reported herein advance the breadth of knowledge regarding Allo’s pleiotropic action in both immature neurons and astrocytes. Allo’s regulation of mitochondrial function in both immature neurons and astrocytes is dependent on both GABA_A_ and L-type calcium activation, suggesting the calcium signaling pathway as a common mechanism of action. The calcium signaling cascade provides a unifying mechanistic pathway that bridges multiple systems of biology in multiple cell types in the brain.

## Data availability statement

The datasets presented in this study can be found in online repositories. The names of the repository/repositories and accession number(s) can be found in the article/supplementary material.

## Ethics statement

All animal studies were performed following National Institutes of Health guidelines on the use of laboratory animals and all procedures were approved by the University of Arizona Institutional Animal Care and Use Committee. The study was conducted in accordance with the local legislation and institutional requirements.

## Author contributions

TW: Conceptualization, Investigation, Writing – original draft, Data curation, Writing – review & editing, Formal analysis. SC: Investigation, Writing – original draft. ZM: Investigation, Writing – original draft. YS: Writing – original draft, Formal analysis. RB: Conceptualization, Supervision, Writing – review & editing, Funding acquisition.
